# Editorial: Novelties in the Therapeutic Approaches for Chronic Heart Failure: Cardiovascular Targets and Beyond

**DOI:** 10.3389/fcvm.2022.895258

**Published:** 2022-07-05

**Authors:** Antonio Sorgente, Andrea Passantino, Massimo Iacoviello

**Affiliations:** ^1^Postgraduate Program in Cardiac Electrophysiology and Pacing, European Reference Networks Guard-Heart, Heart Rhythm Management Center, Universitair Ziekenhuis Brussel-Vrije Universiteit Brussel, Brussels, Belgium; ^2^Department of Cardiology, EpiCURA, Hornu, Belgium; ^3^Department of Cardiology, Istituti Clinici Scientifici Maugeri, Istituto di Ricovero e Cura a Carattere Scientifico (IRCCS), Bari, Italy; ^4^Department of Medical and Surgical Sciences, University of Foggia, Foggia, Italy

**Keywords:** chronic heart failure, vagal nerve stimulation, cardiac resynchronization therapy, relaxin, pulmonary arterial hypertension

Innovation is fundamental in medicine because it enables researchers to overcome the hurdles related to pathologies. This Research Topic was initiated by Iacoviello et al. and published in 2022 in Frontiers in Cardiovascular Medicine. The collection aims to bring together original research or review studies on innovations in the treatment of chronic heart failure (CHF) as a principal aim or target.

*Novelties in heart failure drug therapy*. Over the last few years, a number of new classes of drugs have been demonstrated to further improve HF patients' prognosis and are currently recommended (1). In particular, the “serendipity” of the sodium-glucose cotransporter inhibitors (SGLT2i) changed the paradigm of treatment for patients with HF by demonstrating effectiveness across all the cardiovascular continuum, from diabetic patients at risk of CHF or cardiovascular diseases to diabetic and non-diabetic patients affected by CHF (1). The metanalysis by Zhao L.-M. et al. included the main randomized trials that enrolled patients affected by type 2 diabetes mellitus (T2DM) and evaluated the effects of SGLT2i on major cardiovascular, renal, and heart failure events. The results add new information about the SGLT2i by demonstrating the superiority of the combination of SGLT2i and inhibition of the renin angiotensin system when compared with the inhibition of the renin angiotensin system alone in reducing cardiorenal events.

The effects of SGLT2i are even more striking when the efficacy and safety of SGLT2i in diabetic and non-diabetic patients affected by CHF or chronic kidney disease (CKD) are considered. The metanalysis of Li et al. analyzed data from two main trials evaluating the effects of SGLT2i in patients affected by CHF with reduced ejection fraction (HFrEF), i.e., DAPA-HF and EMPEROR-reduced, and in those affected by CKD, i.e., DAPA-CKD. SGLT2i significantly reduced cardiorenal events, i.e., hospitalization for heart failure and kidney-specific composite outcome, and increased quality of life, as evaluated by Kansas City Cardiomyopathy Questionnaire, without showing any significant increase in events related to adverse effects such as the risk of volume depletion, fracture, amputation, major hypoglycemia, and diabetic ketoacidosis.

Despite the effects of the new pharmacological therapeutic approaches, the prognosis of patients remains poor. For this reason, there is a need to identify new therapeutic targets able to personalize and further improve HF prognosis. The review by Correale et al. shows new perspectives on HF therapy, mainly focusing on new therapeutic targets such as microcirculation, cardiomyocyte, and inflammation.

Besides drug therapy, exercise based cardiac rehabilitation is an approved approach that aims to improve the functional capacity of HF patients. Passantino et al. reported the most recent principles of exercise prescription for CHF patients, which are to obtain meaningful clinical results, without increasing the risk of adverse events.

*The relevance of comorbidities*. Some comorbidities are common in patients with heart failure and affect clinical outcomes. With an elegant experimental design, Hong et al. demonstrated the impact of obstructive sleep apnea on heart function in patients with dilated cardiomyopathy. The suggested mechanism of injury is related to exosomes regulated autophagy (Gong et al.).

The clinical benefit of the treatment of another prevalent comorbidity in CHF is reported by Rizzo et al. The physiopathological, clinical, and prognostic consequences of iron deficiency (ID) are summarized, along with the main results of the FAIR-HF, CONFIRM-HF, and AFFIRM AHF studies, that demonstrated significant improvements in symptoms, quality of life, and clinical outcome after intravenous iron supplementation with ferric carboxymaltose.

ID is now unanimously considered a new therapeutic target in heart failure patients. We cannot overlook the ominous association between heart failure and COVID-19 and have included a paper by Yi et al. that analyzes the association between HF and COVID-19. A deep compression of mechanisms of heart failure in COVID-19 patients is mandatory to identify targets for future therapeutic interventions and reduce the incidence of heart failure and improve outcomes in patients with COVID-19.

*What is new, from the electrophysiology standpoint*. Innovation usually starts from an idea and needs to be tested in an experimental setting. For this reason, we have also included a paper by Zhao S. et al. on “Novelties in the Therapeutic Approaches for Chronic Heart Failure.” Myocardial infarction is one of the most common causes of heart failure: the transition from acute myocardial infarction to chronic heart failure is often unpredictable and is delayed by the established pharmacological and non-pharmacological treatment of heart failure. The authors of this interesting paper focused research efforts on a new non-pharmacological treatment of heart failure, left vagal nerve stimulation. They tested a low level of stimulation, i.e., a level which does not cause significant bradycardia. Routinely used in patients with refractory chronic heart failure as one of the very last therapeutic options (2). The effects of vagal nerve stimulation in the setting of acute myocardial infarction are unknown. The authors decided to set up an experiment in mongrel dogs, seeking to demonstrate the effect of left vagal stimulation in a group of dogs after an iatrogenic myocardial infarction. The primary end points of their research were the incidence of ventricular arrhythmias and the amount of left ventricular remodeling, measured through echocardiographic calculation of left ventricular ejection fraction. Secondary endpoints were evaluation of sympathetic nerve density through immunochemistry of autoptic myocardial tissue and assessment of gene expression profiling through microarray data analysis and quantitative Polymerase Chain Reaction (PCR) analysis. The study used a left vagal nerve stimulation below the threshold, which was used to obtain a bradycardic response in the setting of acute myocardial infarction, demonstrating that it can obtain a significant reduction in the incidence of ventricular arrhythmias and a significant reduction of left ventricular remodeling. Thanks to the immunochemistry data and genetic analysis of the autoptic myocardial tissue, the authors showed that left vagal nerve stimulation can favor cardiac sympathetic neuronal sprouting suppression and decrease myocardial infarction related inflammation reaction. A strength of this study is the presence of two control groups and histological and genetic analysis. The main limitation is intrinsic to all these types of experiments, i.e., myocardial infarction is obtained by the surgical ligature of the left interventricular artery and does not relate to atherosclerosis. Nevertheless, even though it is difficult to predict the reliability of this treatment in the clinical context, it can represent good preliminary data for future clinical trials in the human setting.

Another research study published in this Research Topic, on novel heart failure concerns relaxin (Martin et al.), seems to be a promising innovative treatment for pulmonary hypertension, at least in the experimental context. Martin et al. studied the effect of relaxin on sugen related pulmonary arterial hypertension in a group of rats, with a particular focus on the incidence of ventricular arrhythmia and sinus node dysfunction. The authors demonstrated that relaxin reverses the fibrosis process associated with right ventricular hypertrophy and pulmonary arterial hypertension, which significantly reduced right ventricular remodeling and reduced the incidence of spontaneous and induced life threatening ventricular arrhythmias. The strengths of the present study are the presence of a control group and the use of two different doses of relaxin. The last option furthered understanding by indicating that the effect of relaxin is dose-dependent, since the best effects were obtained when the blood concentrations of relaxin were in the range of 15 ng/mL. Thanks to the immunochemistry results, the authors showed that relaxin favors an increase of beta-catenins at the level of the intercalated disks, favoring better connections of myocardial cells and explaining the higher conduction velocities.

The last and third paper included in the arrhythmology section of the Research Topic concerns the use of a novel pacing algorithm called “SyncAV” (Wang Z. et al.), deployed in patients with CHF receiving an Abbott® cardiac resynchronization therapy device. This algorithm enables it to adapt dynamically to the programmed atrioventricular delay and was demonstrated in combination with biventricular pacing to significantly narrow the QRS duration. The authors sought to investigate the effect of this algorithm on left ventricular remodeling assessed with echocardiography and on NYHA class. One hundred twenty-two patients were consecutively enrolled by Wang Y. et al. and separated into two well-balanced groups according to the use of the mentioned SyncAV algorithm. The patients in whom the SyncAV algorithm was added to the biventricular pacing obtained an acute improvement of the hemodynamics calculated by aortic VTI and subsequently a better echocardiographic and clinical response at mid-term follow-up. The authors should be congratulated for having designed a very simple study with clear endpoints. The paper is an easy read and confirms once more how cardiac resynchronization therapy could be empowered through novel algorithms that allow more physiological pacing. The main limitation of the study was the lack of randomization. The authors overcame this flaw by performing a propensity score matching within the two groups and confirming the original results.

Research in chronic heart failure is a continuous endeavor. We hope to have contributed to these efforts with this Research Topic, opening new treatment pathways that will potentially increase the survival rate of patients with heart failure.

## Author Contributions

All authors listed have made a substantial, direct, and intellectual contribution to the work and approved it for publication.

## Conflict of Interest

The authors declare that the research was conducted in the absence of any commercial or financial relationships that could be construed as a potential conflict of interest.

## Publisher's Note

All claims expressed in this article are solely those of the authors and do not necessarily represent those of their affiliated organizations, or those of the publisher, the editors and the reviewers. Any product that may be evaluated in this article, or claim that may be made by its manufacturer, is not guaranteed or endorsed by the publisher.
